# Challenges Associated With the Civilian Reintegration of Soldiers With Chronic PTSD: A New Approach Integrating Psychological Resources and Values in Action Reappropriation

**DOI:** 10.3389/fpsyt.2018.00737

**Published:** 2019-01-08

**Authors:** Célia Belrose, Anais M. Duffaud, Frédéric Dutheil, Julie Trichereau, Marion Trousselard

**Affiliations:** ^1^Unité de Neurophysiologie du Stress, Département de Neurosciences et Sciences Cognitives, Institut de Recherche Biomédicale des Armées, Brétigny sur Orge, France; ^2^APEMAC, EA 4360, EPSaM, Université de Lorraine, Lorraine, France; ^3^Faculty of Health, Australian Catholic University, Melbourne, VIC, Australia; ^4^Department/Laboratory: Physiological and Psychosocial Stress, Université Clermont Auvergne, LaPSCo, CNRS, Clermont-Ferrand, France; ^5^Ecole du Val de Grâce, Paris, France

**Keywords:** recovery, reintegration, positive psychology, post-traumatic stress disorder, quality of life, mental illness, values in action, military

## Abstract

**Background:** In light of the psychological changes in an individual suffering from chronic Posttraumatic Stress Disorder (PTSD), questions are being raised in order to understand and facilitate recovery and a return to work. This is particularly challenging for soldiers suffering from chronic PTSD, who are often young individuals suffering from moral conflicts. A French military rehabilitation program proposes the broadening of the relationships between recovery and reintegration by incorporating approaches from the field of positive psychology for soldiers with chronic PTSD. The aim of the study was to evaluate (i) the psychological resources which remain sustainable for these trauma exposed soldiers according to their PTSD symptoms, (ii) the dynamics of resource reappropriation after the military rehabilitation program, which focuses on values in action (VIA) as character strengths, and (iii) how these resources and their reappropriation facilitate civilian professional reintegration.

**Method:** We conducted a prospective study with 56 trauma exposed soldiers with a clinical diagnosis of chronic PTSD. PTSD severity and psychological resources (optimism, mindfulness, well-being, motivation, self-esteem, and VIA) were assessed before and after the rehabilitation program. After the identification of resource profiles, we analyzed the impact of the program on resource levels and successful reintegration into a civilian job.

**Results:** 3 profiles were identified based on the psychological resources of the soldiers. Profiles 1, 2, and 3 differed in terms of clinical severity (PCL5). Profile 1 exhibited both the highest level of resources and the lowest clinical severity of PTSD but did not modify its resources after the intervention program when compared to profile 3. Profile 3 was characterized by the lowest level of resources, the highest clinical severity of PTSD and the highest reappropriation in all VIAs. This profile was associated with the highest rate of reintegration success 1 year after the intervention.

**Conclusions:** This paper aims to broaden the relationship between recovery and reintegration by incorporating approaches from the field of positive psychology for soldiers with PTSD. VIA appears to be an important factor for reintegration. Our results highlight the importance of taking into account the existing needs of the patient and the optimization of the modalities of individual, collective, and institutional rehabilitation for patients suffering from PTSD in order to better understand the dynamics of the recovery process of a chronically afflicted individual.

## Introduction

### Post-traumatic Stress Disorder (PTSD)

PTSD is a debilitating mental disorder that may develop after experiencing or witnessing a life-threatening event. The main characteristics of PTSD are re-experiencing symptoms, avoiding situations that recall the event, increased negative beliefs and feelings and hyperarousal (1). This suffering is associated with impairment in social, occupational and other domains (2). Furthermore, strong associations are commonly described between PTSD and comorbid conditions, which include: depression, substance use disorders, and general physical health effects (3, 4). To be diagnosed with PTSD, a person must experience those symptoms for at least 1 month. Once the symptoms have been observed for 3 months, PTSD is considered as a chronic disorder (5).

PTSD has a prevalence ranging from 1 to 7% in Europe (4). A clinical review on PTSD (6) mentioned how “*emphasis is being placed on identifying factors that explain individual differences in responses to trauma and promotion of resilience."* It also mentioned a higher prevalence, ranging from 25 to 50% depending on the type of trauma. PTSD prevalence in military settings is highly dependent on the violence of the mission; the higher the combat exposure, the higher prevalence of PTSD (up to 20%) (7).

With appropriate care, treatment efficiency is variable and around 20% of the patients do not respond to psychological treatment (Nice 2016). On the one hand there is little research to indicate which treatments are most effective for which patients. On the other hand, a 20-year longitudinal study on a cohort of 214 veterans showed how initial combat stress reaction could lead to volatile chronic stress, with ~40% of recovering subjects relapsing within 1 year of remission (8).

Unresolved, PTSD can become chronic, causing anguish and suffering in the primary victim and their loved ones. Due to the relatively high prevalence of PTSD in the population, particularly the military, there is an urgent need for treatments that effectively improves recovery. Such statements imply the establishment of an integrated system for the intake of chronic PTSD patients and the evaluation of its impact on the usual impairments of occupational and academic functioning (9–12), marital and family functioning (11, 13, 14), parenting (15, 16), and friendships and socializing (17).

Such impairments are common among military personnel deployed in combat overseas, and particularly among cases of chronic military PTSD (11, 18–20). They contribute to homelessness and unemployment (21, 22). This point is all the more important for French soldiers with chronic PTSD, who have to leave the military institution when their authorized sick leave is over, which can be between 3 and 8 years depending on their military status.

### French Military Rehabilitation Intervention Management

**Figure 1:**
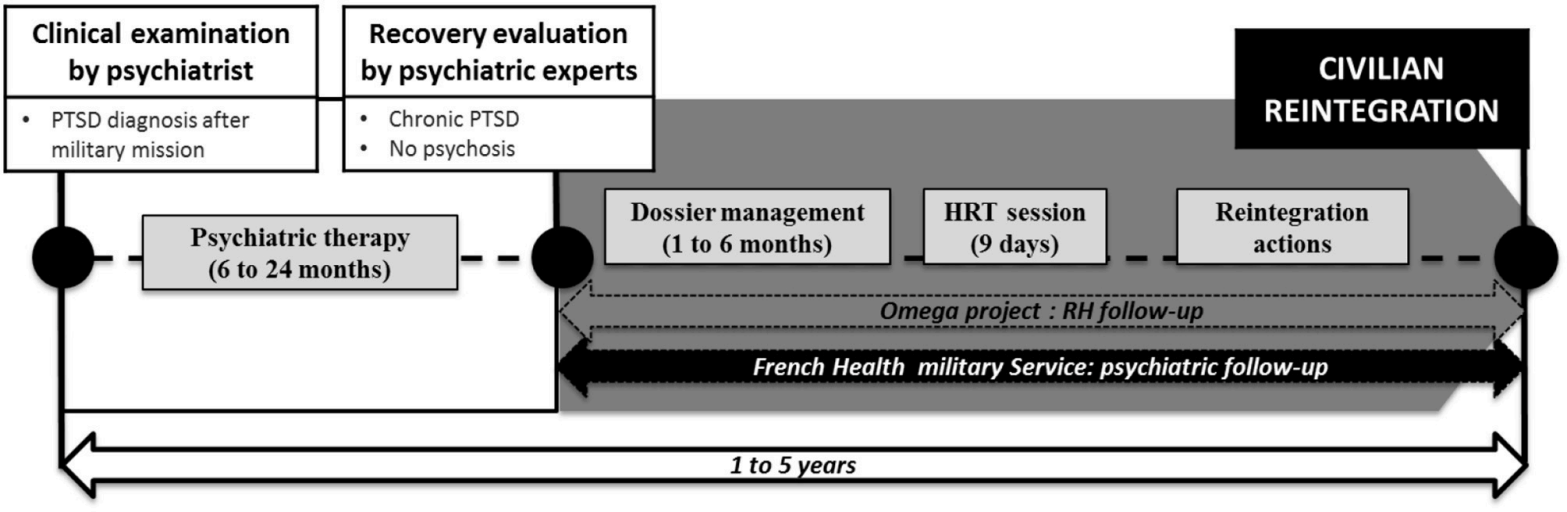
French military rehabilitation for PTSD from diagnosis to reinsertion: position of the *omega* project. HRT: Human Resources Training.

In 2016, the French Army developed a rehabilitation intervention program to help the trauma exposed soldiers with a clinical diagnosis of chronic PTSD. This program is coordinated by a specific Army office called the *Cellule d'Aide aux Blessés de l'Armée de Terre* (CABAT; support office for wounded soldiers). This so-called *omega* project is based on an integrative program using psychosocial interventions, sporting activities, coaching, and human resource supports. It also provides administrative support and legal advice. Some civilian partner companies participate in the *omega* project. To enter the omega program, a soldier must be referred by the French Military Health Service after approval by a psychiatrist (Figure 1). Soldiers exhibiting complex PTSD or psychosis after psychiatric examination were not included in the *omega* project.

**Figure 2:**
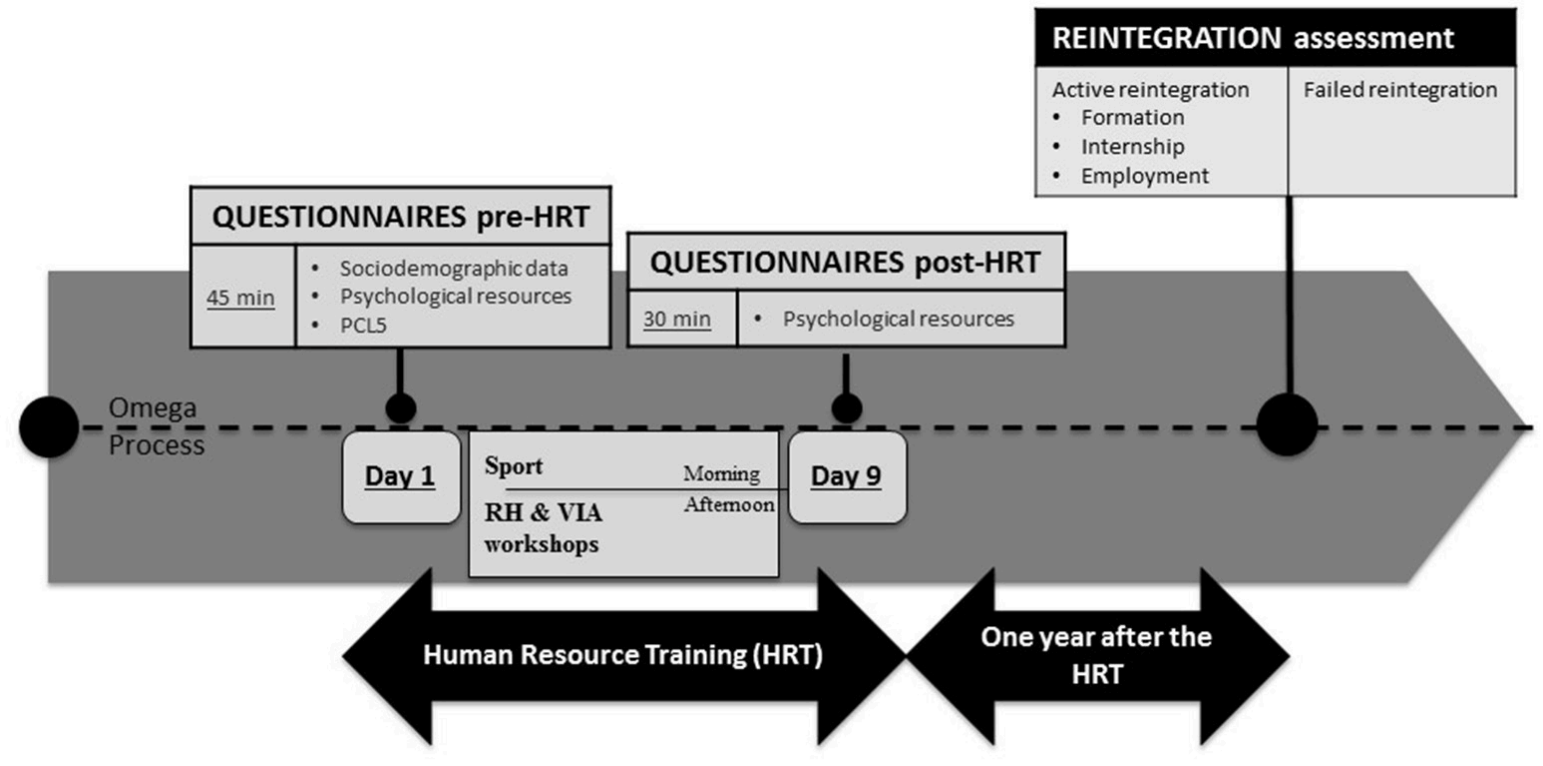
Experimental protocol with the distribution of the three sessions: first day of the Human Resource Training (pre-HRT), the last day of the HRT (post-HRT), and 1 year after.

The project began with a 9-days training session (called Human Resources Training—HRT). The HRT took place in a natural environment that focused on body reappropriation (with daily sporting activities), coaching and human resources practical workshops, and group challenges (Figure 2). Ten to 15 people with chronic PTSD were included in each HRT session. After the 9-days training session, each person had an individual professional reintegration project plan. This professional project plan included an outline of the scope of the plan and its objectives, as well as key milestones. In order to succeed in this process, each participant had a monthly follow-up to examine administrative and legal needs. They also received guidance in order to correctly settle in to their new professional environment (either in partner companies or in others companies of interest for the individual reintegration project). They also continued to have a monthly psychiatric follow-up.

In order to help trauma exposed soldiers with chronic PTSD to deal with daily worries and stress during their reintegration program, two main positive psychology approaches were used during the 9-days training program. The first consisted of focusing the attention (daily 5-min exercises and verbal reinforcement of appropriate behaviors) on psychological resources such as self-esteem, optimism, mindfulness and mind-body connections, openness, attention to the natural environment, and team building. The second focused on personal strengths and values related to the main objective in order to help them to reappropriate their own strengths and values (23). For this, they attended a curriculum vitae workshop based on the narrative of military actions to highlight military and civilian competences as well as to detect moral conflicts that they could have faced during military deployment. Moral conflicts are known to induce moral stress by acting in conflict with one's own conscience, e.g., when one knows the right thing to do but institutional constraints make it difficult to act in a way that is consistent with one's morals (24). The workshop is face-to-face and can be divided into several sessions over the 9-days training period. Thus, at the end of the workshop each participant has a civilian curriculum vitae and has also identified which values in actions (VIA) are important for them (using the narrative reappropriation of their military acts in their personal history).

In the next step of the reintegration process, individual VIAs were taken into account in order to choose either immersion or reintegration into a company that matched those values, so as to avoid any conflicts between company values, micromanagement values, and personal values. This is important because conflict situations can induce moral stress, which is a risk factor for relapse (25).

To face the challenges of recovery and reintegration into civilian society for trauma exposed soldiers with chronic PTSD, we conducted a prospective study to assess the relationship between psychological resources and chronic PTSD profiles. We aimed to evaluate (i) which resources remain sustainable based on the chronic PTSD profiles, (ii) the dynamics of resource reappropriation after the military rehabilitation program (focusing on VIA as character strengths), and (iii) whether or not resources and their reappropriation facilitate civilian professional reintegration.

## Materials and Methods

### Participants

Sixty voluntary French trauma exposed soldiers with chronic PTSD related to military deployment were included in a prospective study. Diagnosis was established through a clinical psychiatric exam conducted by a military psychiatrist. They were recruited from within the CABAT process after psychiatric therapy, when the psychiatrist considered the recovery appropriate enough to take part in the *omega* project. All soldiers included in this study were on sick leave for at least 6 months due to chronic PTSD and were waiting for a military invalidity committee pension. They were all engaged in the first step of the *Omega* project and had a 9-days HRT training session planned.

Data collection was carried out during 6 HRT programs between May 2016 and May 2017. Four individuals were excluded from the analysis due to missing data (they did not complete the questionnaires). This study received the agreement of the ethics committee of the French military health service. All the subjects received information on the protocol and gave their written consent prior to their participation.

### The HRT Program

A HRT was organized for a group of 10 participants. It took place in “les Ecrins National park” in a small village in the countryside. Each morning, from 9 a.m. to 12 p.m., participants had sporting activities: mountain walking, mountain biking, climbing, canyoning, and collective orienteering running. From 3 p.m., they had individual and collective practical workshops based on PTSD psychoeducation, human resources competences and coaching, including a curriculum vitae workshop. During their free time, they could take part in collective activities like table football, pool, party games, or have a rest. Relaxation exercises were proposed every day after the sporting activity and before dinner. During the last 2 days of the HRT, families were invited to share a mountain walk and have psychoeducation on PTSD and its evolution.

Civilian or military experts in human resources and social reintegration conducted the workshops. A military psychologist was present throughout the HRT in order to provide support for anxiety or symptoms of substance dependence symptoms. None of the subjects left the HRT.

### Psychological Variables

The following socio-demographic data were collected: age, gender, social environment, and the number of major stresses encountered in professional and personal environments over the patient's life (Table 1).

**Table 1:** Sociodemographic characteristics of the subjects.

**Gender**	
Male (%)	53 (94.64)
Female (%)	3 (5.36)
**Mean age in years (standard deviation)**	34.5 (±9.3)
**Marital status**	
Living with a partner (%)	35 (62.5)
Single (%)	21 (37.5)
**Military seniority (years)**	13 (±8.6)
**Military rank (%)**	
Soldiers	20 (35.71)
Non-commissioned officers	33 (58.93)
Officers	3 (5.36)
**Reported stressful event throughout the patient's life (%)**	
Yes (%)	2 (3.54)
No (%)	54 (96.46)
**Traumatic military deployment (%)**	
Yugoslavia	3 (5.36)
Afghanistan	32 (57.14)
Mali	12 (21.43)
Central Africa	9 (16.07)
**Sick leave duration (months)**	22 (±7.4)

#### Psychological Functioning

Among the 10 self-reporting questionnaires evaluating psychological functioning, one focused on the severity of the PTSD, one on self-esteem, four on psychological resources (optimism, motivation and the Life Orientation Test–Revised), two on body-mind connection (mindfulness and body consciousness), one on VIA, and one on well-being.

The questionnaire used for PTSD severity was the PTSD Check List Scale (PCL-5) (1, 26). It assesses the following four symptoms: re-experiencing symptoms, avoiding situations that recall the event, hyperarousal, and impairment of cognitive and emotional functioning. Two cutoff points have been proposed by the National Center for PTSD: above or equal to 33 or above or equal to 38. We chose the highest of these two thresholds, which have previously been proposed in the literature, in order to increase the specificity of our PTSD diagnosis (26, 27). Individuals with a threshold above or equal to 38 were then considered as suffering from PTSD.

Self-esteem was measured using the Rosenberg Self Esteem Scale (SES) (28). This 10-item self-assessment scale evaluates overall self-worth by measuring both positive and negative feelings about self. All items are answered using a 4-point Likert scale ranging from (1) “totally disagree” to (4) “totally agree.” A score below 15 is considered as a low self-esteem whereas a score between 15 and 25 is considered as a normal self-esteem.

The Orientation to Happiness questionnaire [OTH, (29, 30)] is an 18-item self-report assessment with six items for each of the three dimensions: hedonism/pleasure, engagement, and meaning of life (29). The 12 items in the meaning of life and pleasure dimensions are in line with the hedonic vs. eudemonic conceptions of happiness observed in previous research (29). The remaining six items, which measure engagement, are based on the work by Csikszentmihalyi (31) and characterize the “flow” state of absorption in a task. The responses are given on a five-point Likert scale ranging from (1) “very much unlike me” to (5) “very much like me.”

The Global Motivation Scale [GMS, Guay et al. unpublished manuscript] measures the overall motivation that people have to do things in their life. The Motivation scale appoints “a hypothetical intra-individual strength, which can have multiple internal and/or external determiners, and which helps to explain the direction, the release, the obstinacy and the intensity of the behavior or the action” (32). Motivation is a multidimensional concept, which is explained by the continuum of the Self-Determination Theory (SDT). SDT proposes three categories of motivation in this continuum of self-determination (33): the intrinsic motivation (IM), the extrinsic motivation (EM), and the amotivation (AM). Intrinsic motivation represents the highest level of self-determination, while the amotivation corresponds to a deficit of self-determination. The three basic psychological needs of the SDT are: competence (32), relatedness (34, 35), and autonomy (33). The GMS assesses three sub-factors of intrinsic motivation (knowledge, stimulation and accomplishment) (36–38), three sub-factors of extrinsic motivation (identified, introjected, and external regulation), and amotivation. There are 28 items, i.e., 4 for each of the 7 sub-scales. Every statement is measured on a Lickert scale ranging from (1) “does not correspond at all” to (7) “corresponds completely.”

The Life Orientation Test–Revised [LOT-R; (39, 40)] evaluates the dispositional optimism. The LOT-R is a 6-item self-report measure (with four filler items) that evaluates the respondent's generalized expectations of positive (three items) and negative (three items) outcomes. For each item, the subject has to indicate if it characterizes their feelings using a 5-point Likert scale ranging from (0) “totally disagree” to (4) “totally agree.”

The Freiburg Mindfulness Inventory-14 (FMI) is a short form of 14 items developed for people without any background knowledge of mindfulness (41, 42). It constitutes a consistent and reliable scale evaluating the state of mindfulness and two subfactors (43): “acceptance” as an ability to embrace unwanted thoughts and “feelings” as an alternative to experiential avoidance and being present, which characterizes being in non-judgmental contact with environmental events as they occur. Each self-descriptive statement is evaluated using a 4-point Likert scale ranging from (1) “strongly disagree” to (4) “strongly agree.”

The Body Connection Scale [BCS, (44)] is a 20-item scale designed to assess body awareness with a two-faceted sensory awareness and bodily dissociation. Sensory awareness evaluates the ability to identify and experience inner sensations of the body and the overall emotional/physiologic state of the body, such as bodily changes/responses to emotions and/or the environment (12 items). The concept of bodily dissociation is characterized by the avoidance of internal experience. Bodily dissociation has experiential aspects, including normal everyday experiences, e.g., distraction from bodily experience or emotional disconnection (8 items). Each self-descriptive statement is evaluated using a 4-point Likert scale ranging from (0) “strongly disagree” to (3) “strongly agree.”

The Warwick-Edinburgh Mental Well-Being Scale [WEMWBS; (45, 46)] covers both affective constructs (including the experience of happiness) and constructs representing psychological functioning and self-realization (47). This is a 14-item scale on thoughts and feelings over the past week; each item ranges from (1) “none of the time” to (5) “all of the time.”

The Values in Action Inventory of Strengths (VIA-IS) is a self-report assessment designed to identify an individual's possession of 24 character strengths. Those character strengths contribute to the six main VIA (23): wisdom, courage, humanity, temperance, justice, and transcendence. We used the French 24-item brief scale of the Values in Action (48). The subject was asked to rate each of the 24 items on a 5- scale point Likert (“1 = very much unlike me” through “5 = very much like me”).

#### Reintegration Assessment

In agreement with the officer directing the CABAT, 1-year reintegration success was evaluated. Each subject was classified in either the “active reintegration” group (AR) or in the “failed reintegration” group (FR). The AR group corresponded to subjects who managed to undergo a formation related to their professional objective or to have correctly settled in to a chosen company (internship or employment). The FR group included subjects who did not reach one of these two professional objectives.

### Experimental Procedure

Each HRT started on a Saturday. Figure 2 sums up the experimental protocol. There were two evaluation sessions during the HRT: pre-HRT session (first day) and post-HRT (last-day) and a third session 1-year after the HRT program with the director of CABAT office.

### Statistical Analysis

The data were recorded in Excel 2010 (Microsoft®, Redmond, WA, USA) and analyzed with Stata® V13 (StataCorp LP, texas, USA).

To assess the relationship between psychological resources and chronic PTSD, a mixed Ascending Hierarchical Classification was applied to first identify psychological profiles from the pre-HRT session variables (SES, optimism, motivation, LOT, FMI, BCS, WEMWBS, and values in action). The differences in the PCL-5 scores (and sub-factors) between the profiles were assessed using an Analysis of Variance followed by Newman-Keuls's *post-hoc* test in order to determine if the differences were statistically significant. Fisher exact tests were applied to compare the percentage of subjects with a PTSD between the profiles.

To evaluate the dynamics of resource reappropriation after the military rehabilitation program, score variations (between pre- and post-HRT) were calculated for each of the recorded variables (SES, optimism, motivation, LOT, FMI, BCS, WEMWBS, and VAI). A percentage change of more than 20% was considered as a relevant/significant change, meaning either a relevant/significant decrease for scores that were reduced after the HRT or a relevant/significant increase for scores that were improved after the HRT.

Whether or not the resources and their reappropriation facilitated civilian professional reintegration was assessed using Fisher exact tests to compare changes between the three profiles and to evaluate the impact of the HRT on the success of reintegration based on the chronic PTSD profile at the pre-HRT session.

In all cases, a difference was considered significant when *p* < 0.05.

## Results

### Study Population

Table 1 summarizes the bio-demographic characteristics of the 56 subjects.

71.43% exhibited a PCL5 score above 38 (M 45.8 ± 15.3). No differences were found in the PCL5 score according to gender, marital status, military rank, or deployment where the trauma occurred.

### Psychological Profiles

Three profiles were identified by the psychological functioning assessment performed on the first day of the HRT: profile 1 consisted of 32 subjects, profile 2 consisted of 10 subjects and profile 3 consisted of 14 subjects. Table 2 sums up the psychological functioning for each of the three profiles.

**Table 2:** Psychological scores in the pre-HRT session according to the three profiles obtained by the mix Ascending Hierarchical Classification.

		**Profiles**		
		**P 1**	**P 2**	**P 3**
		***n* =32 (57.14%)**	***n* = 10 (17.86%)**	***n* = 14 (25%)**
		**M (± SD)**	**M (± SD)**	**M (± SD)**
Self-esteem		29.1 (±4.76)	22.6 (±2.68)	21.7 (±3.60)
Orientation to happiness	Hedonism/pleasure	22.6 (±3.24)	20.5 (±1.17)	20.1 (±3.76)
	Engagement	17.2 (±3.05)	15 (±3.46)	13.8 (±4.29)
	Meaning of life	28.1 (±3.32)	24.8 (±3.45)	24 (±4.45)
Motivation	IM Knowledge	23.2 (±2.88)	16 (±2.66)	17.1 (±6.15)
	IM Accomplishment	22.5 (±3.82)	17.8 (±3.64)	16.9 (±6.08)
	IM Stimulation	21.3 (±4.36)	15.8 (±1.93)	16.2 (±5.25)
	EM Identified	20.3 (±3.87)	16.8 (±3.01)	13.6 (±4.76)
	EM Introjected	16.5 (±6.04)	15.1 (±4.77)	15 (±5.30)
	External regulation	15.1 (±5.23)	13.3 (±4.24)	14 (±3.63)
	Amotivation	11.4 (±5.38)	11.7 (±2.86)	14 (±5.18)
Optimism		22.5 (±2.83)	16.8 (±2.58)	19.6 (±3.41)
Mind-fullness	Total	36.3 (±6.46)	26.2 (±4.13)	25.3 (±3.19+)
	Presence	16.7 (±3.46)	11.7 (±2.66)	12.2 (±3.11)
	Acceptation	19.6 (±3.53)	14.5 (±1.95)	13 (±2.20)
Body connection	Body awareness	21.5 (±5.56)	29.1 (±6.65)	19.7 (±6.85)
	Bodily dissociation	15.3 (±5.01)	17.1 (±4.10)	12.3 (±5.96)
Well-being		46.2 (±8.83)	34.3 (±4.82)	31.6 (±7.45)
VIA	Wisdom	3.4 (±0.77)	2.8 (±0.51)	1.6 (±0.76)
	Courage	3.5 (±0.78)	3.6 (±0.72)	1.7 (±0.91)
	Humanity	3.4 (±0.74)	3 (±0.79)	1.9 (±0.93)
	Justice	3.4 (±1.36)	3.9 (±0.66)	1.3 (±1.06)
	Temperance	3.2 (±0.73)	2.8 (± 0.74)	2.1 (±0.87)
	Transcendence	3.1 (±0.76)	2.9 (±0.32)	1.6 (±0.75)

*P, profile; n, number; M(±SD), mean (±standard deviation)*.

As described in Table 3, total PCL5 score and sub-factor scores were different between the profiles (Table 3). Profile 1 has a lower PLC-5 score (and sub-factors) than profiles 2 and 3. Differences between profiles 2 and 3 were only observed for intrusive symptoms with the highest score being for profile 2. The number of subjects with PTSD, defined by a PCL5 score above the threshold of 38, was different between the profiles (Chi^2^ = 9.01; *p* = 0.012) with 16 subjects (50%) in profile 1, 10 subjects (100%) in profile 2, and 12 subjects (85.71%) in profile 3.

**Table 3:** PCL5 scores in the pre-HRT session according to the three profiles.

		**Profile 1 *n* = 32 (57.14%) M (±SD)**	**Profile 2 *n* = 10 (17.86%) M (±SD)**	**Profile 3 *n* = 14 (25%) M (±SD)**	**p**
PCL5	PCL 5 TOTAL	37.8 (±17.56)	56.9 (±10.18)	50.1 (±14.16)	*p* = 0.003
	PCL (Repetition)	10.1 (±5.8)	15.2 (±3.33)	11.5 (±4.93)	*p* = 0.042
	PCL (Avoidance)	8.25 (±3.97)	12.2 (±2.53)	10.57 (±3.88)	*p* = 0.012
	PCL (Hyperarousal)	9.4 (±5.3)	13.8 (±2.82)	13 (±4.13)	*p* = 0.016
	PCL (cognitive and emotional dysfunctions)	10.06 (±5.13)	15.7 (±2.66)	15 (±4.04)	*p* = 0.006

*N = number; M(±SD): mean (±standard deviation); M, mean; SD, standard deviation*.

### Impact of the HRT on Psychological Functioning According to the Profiles

No difference was observed between the three profiles in terms of the percentage of subjects exhibiting a significant (20%) change in the scores for the following psychological resources: well-being, body awareness, and bodily dissociation, and orientation to happiness (and sub-factors).

Table 4 summarizes the psychological resources with a pertinent change after the HRT, which differed between profiles in the percentage of subjects. Significant differences in the percentage of subjects exhibiting pertinent changes were observed for mindfulness, optimism, self-esteem, and both wisdom and courage VIA. There was a tendency for differences in the percentage of subjects with pertinent changes for justice, temperance, and transcendence VIA.

**Table 4:** Percentage of subjects in each profile for the psychological resources with significant changes at the post-HRT compared to the pre-HRT session.

		**Profile 1**			**Profile 2**			**Profile 3**			***p***
		**= (%)**	**↗ (%)**	**↘ (%)**	**=(%)**	**↗ (%)**	**↘ (%)**	**= (%)**	**↗ (%)**	**↘ (%)**	
Mindfulness		52.4	19	28	28.6	71.4	0	44.4	55.5	0	0.04
Optimism		92.3	7.7	0	33.3	66.7	0	60	40	0	0.02
Self-esteem		87.5	4.2	8.3	50	37.5	12	66.7	33.3	0	0.04
VIA	Wisdom	72	24	4	37.5	37	25	11.1	88	0	0.01
	Courage	60	28	12	62.5	0	37	33.3	66	0	0.02
	Justice	69.6	13	17.4	66.7	0	33.3	28.6	57.1	14.3	0.06
	Temperance	56.5	21.7	21.7	66.7	16.7	16.7	12.5	75	12	0.07
	Transcendence	58.3	20.8	20.8	83.3	0	16.7	37.5	62	0	0.08

* = , no change above 20%; ↗ : increase in the score of more than 20%; ↘ : decreased score of more than 20%*.

When regarding pertinent decreases in psychological resources after the HRT, we observed that, whatever the profile, no subject experienced a decrease in the optimism resource after the HRT program. No subject in profile 3 had a decrease >20% after the HRT program for mindfulness, self-esteem, optimism, and wisdom, courage, and transcendence VIA. However, a decrease of more than 20% was observed for justice and temperance VIA.

### Impact of the Profiles on Reintegration Success at 1 Year

Differences were observed between profiles in terms of reintegration (Chi^2^ = 8.03; *p* = 0.03), with more profile 3 subjects in AR. For profile 1, 60% were categorized as FR and 40% in AR; for profile 2, 80% were categorized as FR and 20% as AR; and for profile 3, 28.6% were categorized as FR and 71.4% as AR.

## Discussion

This study focused on the challenges of recovery and reintegration into civilian society for soldiers with a clinical diagnosis of chronic PTSD due to trauma experienced during overseas deployments. Firstly, at the beginning of the HRT program, results showed different profiles of sustainable psychological resources. According to the hierarchical classification of the results, three profiles were identified in terms of mindfulness and body connection, optimism, orientation to happiness, motivation, well-being, and VIA. The first profile exhibited the highest resources compared to the other two, particularly in terms of mindfulness, optimism, self-esteem and values in action. Profile 2 and profile 3 were different in terms of VIA, with the lowest levels for wisdom, courage and justice observed in profile 3 compared to profile 2. The lowest PTSD symptoms, irrespective of the symptom clusters, were observed for profile 1. Differences between profiles 2 and 3 were only observed for intrusive symptoms, with the highest score for profile 2. Although all soldiers included in the study were on sick leave for at least 6 months because of chronic PTSD, around 29% did not have a PCL5 score above the threshold at the beginning of the HRT. The number of soldiers with a psychometric diagnosis of PTSD was different between profiles; with the lowest number for profile 1 compared to profiles 2 and 3. The differences between the profiles must be taken into account as PTSD is not only a fluctuant disorder with periods of remission, but is also volatile from 1 day to another in terms of symptoms (8). Moreover, inter individual differences in the efficiency of the psychiatric therapy must be taken into account when considering the discordance between subjects. Altogether, these results showed that the lowest clinical suffering was associated with the highest sustainable psychological resources for soldiers with chronic PTSD (profile 1). Furthermore, differences in terms of VIA were found in the high clinical suffering profiles (profile 2 and profile 3), with profile 2 exhibiting higher levels of VIA than profile 3. Taken together, these statements demonstrate that psychological functioning is not systematically linked with the severity of the disorder in soldiers with chronic PTSD. This must be taken into account when customizing the recovery management program for chronic PTSD to the patient's available psychological resources.

Secondly, we observed different resource reappropriation dynamics after the military HRT program. Resource reappropriation was defined as an increase of more than 20% in the score at the end of the HRT program. Differences between the profiles were observed in resource reappropriation for mindfulness, self-esteem, optimism, and values in action, and in particular for wisdom, courage, and justice; with the highest reappropriations for profile 3. Interestingly, except for justice and temperance VIA, subjects in profile 3 exhibited no pertinent decreases in psychological resources in post-HRT, whereas this profile was characterized by low scores in VIA at pre-HRT program. Two other results should be highlighted. The first is that, whatever the profile, optimism was not associated with a pertinent decrease after the HRT program. The second is that no pertinent changes were observed between the profiles for motivation. One explanation for this latter could be that a scale focusing on general motivation assessed motivation. The assessment of motivation to a specific task (reintegration program, physical activities, or practical workshop) could provide different results.

Finally, reintegration success 1-year post HRT was different between profiles. A higher percentage of reintegration success was observed for profile 3, which was characterized on the one hand by a low level of resources associated with a high level of PTSD severity at the beginning of the HRT, and on the other hand by a higher number of subjects with resource reappropriation after the HRT. These results suggest that reappropriation of resources, particularly VIA, facilitate civilian professional reintegration for military personnel with chronic PTSD. These exploratory data, focusing on psychological positive resources and PTSD reintegration, showed that (i) psychological functioning is affected in different ways in chronic PTSD depending on the soldier, (ii) successful reintegration was associated with the ability to reconnect with personal resources, and (iii) soldiers who reconnected themselves were not suffering less.

Altogether, these results raise several questions. The first concerns the interindividual differences for the resource reappropriation. The assessed psychological resources belong to positive psychology, which is “the study of the conditions and processes that contribute to the flourishing or optimal functioning of people, groups, and institutions” (49). Specifically, positive psychology grew largely from the recognition of an imbalance in clinical psychology, where most research focuses on mental illness. From its individual point of view, positive psychology focuses on understanding how human strengths can lessen the damage of disease, stress and disorder. It defines Post-Traumatic Growth (PTG) as “the experience of positive change that occurs as a result of the struggle with highly challenging life crises. It is manifested in a variety of ways, including an increased appreciation for life in general, more meaningful interpersonal relationships, an increased sense of personal strength, changed priorities, and a richer existential and spiritual life” (50). PTG is supposed to emerge from cognitive processes and can have functional and dysfunctional aspects. How PTG is related to specific psychological variables and if there are biological variables linked to PTG is still poorly understood. A recent systematic review of the literature indicated that trauma survivors with PTSD exhibit more PTG than those without PTSD and that PTG can be intensified during the therapeutic process (51). Whether positive correlations between PTG and PTSD are reported, no evidence of a quadratic relationship between PTG and PTSD was found. Results of two studies suggest that when the level of post-traumatic stress reaches the threshold of the diagnostic criteria, the momentum for growth seems to be hindered (52). Moreover, findings regarding the association of PTG with psychological variables are mixed (51). However, Calhoun suggests that a form of wisdom occurs for most patients with PTSD (53). Calhoun showed that people who have dealt with major events develop specific skills such as: an ability to balance between reflection and action; an ability to weigh the known and the unknown; a better ability to accept the paradoxes of life; and a better ability to ask the fundamental questions of their existence in a more open and satisfying way. Whether reintegration is considered as applied PTG or not, our results suggest that for the profile 3, which exhibited high levels of suffering, PTG was reached through resource reappropriation, and that the HRT helped as a generator of internal resources, particularly VIA.

A second question concerns the importance of VIA in the success of the reintegration program. Little information can be found in the literature about the prognostic relationship between VIA and PTSD severity. Psychiatric classification primarily describes negative aspects of a patient's life and provides little information on a patient's psychological strengths, such as the values in action (54). Recently, moral injury has appeared in the spectrum of PTSD, defined as a syndrome characterized by psychological and religious/spiritual symptoms of inner conflict. The presence of psychological or religious/spiritual symptoms may depend on whether the service member is spiritual and religious or spiritual but not religious, or neither (55). Litz proposed that moral injury occurs when “perpetrating, failing to prevent, bearing witness to, or learning about acts that transgress deeply held moral beliefs” (56). Moral injury can lead to experiencing “a deep sense of transgression, including feelings of shame, grief, meaninglessness, and remorse from having violated core moral beliefs” (57). Individuals suffering from moral injury appear to struggle with their personal values. In line with this, one hypothesis is that our profile 3 patients may suffer from moral injury. Further research is needed to assess this proposition and to evaluate whether the HRT program is an efficient intervention to decrease symptoms with respect to moral injury confrontation.

The third question constitutes a more applied question and focuses on the best way to provide recovery in chronic PTSD, with reintegration being one of the recovery dimensions. The HRT program failed to reintegrate around half of the soldiers with chronic PTSD 1 year after the program. It is important to identify soldiers who are not helped by the care in order to improve the practical workshops and follow-up that exists in the omega process. Nevertheless, it constitutes an original care management approach for recovery by promoting a salutogenic approach which is based on positive mental health (58). Its aim is to characterize the condition of the sick individual on a positive mental health continuum, which is part of the comprehensive mental health (59). Thus, the salutogenic approach is complementary to the usual pathogenic vision, which is oriented on clinical result “indicators directly and only associated with the disease.” A systemic approach for recovery and reintegration should involve general psychiatric rehabilitation (pathogenic approach) that integrates, in its interventions, some tools aimed at “spreading and developing” positive resources which are part of positive psychology (salutogenic approach) (60). The HRT program is in line with these positive integrated approaches of recovery. It needs to be refined to improve its benefits on recovery and reintegration. For instance, some improvements of the HRT program may focus on cognitive and emotional remediation exercises (mind-body connection interventions).

This study has shown several limitations. Firstly, diagnosis was established through a clinical exam conducted by a military psychiatrist before inclusion into the *omega* project. In this study, status related to PTSD were assessed using the PCL5 and 29% of these soldiers did not have a PCL5 score above the threshold at the beginning of the HRT. The use of the gold standard assessment [Clinician-Administered PTSD Scale for DSM-5; (61)], which is not yet validated in French, would refined and confirm or not the PTSD diagnosis at the beginning of the HRT. Secondly, the study included mainly male subjects, thus requiring the reproduction of these results in a female population. This is all the more important as gender difference is well-described in PTSD (6). Thirdly, the sustainability of reintegration was not evaluated. Relapses are frequent for chronic PTSD (8) and knowing how soldiers who were reintegrated into civilian jobs deal with daily stress is important to improve their remission. Fourthly, we did not evaluate moral injury, which appears to be an important factor in understanding recovery from chronic PTSD (62). Whether the HRT program is efficient for chronic PTSD with moral injury cannot really be evaluated. Finally, no information was available about which practical workshop in the HRT program was the most effective for resources, VIA, and reappropriation.

## Conclusion

In this paper, we propose the broadening of the relationships between recovery and reintegration in PTSD by integrating approaches from the field of positive psychology. Positive resources that are still available are linked to clinical severity despite the psychic wounds of the soldiers. Some resources (i.e., VIA, mindfulness, optimism, etc.) appear to be more efficient in helping the reintegration of soldiers with chronic PTSD. Altogether, our results highlight the importance of taking into account the existing needs of the patient and optimizing the modalities of individual, collective and institutional rehabilitation of individuals suffering from PTSD in order to better understand the dynamics of the recovery process. This suggests that future programs focusing on salutogenic recovery interventions and reintegration for traumatized soldiers could develop and validate more practical workshops for improving resources, particularly VIA.

## Author Contributions

CB and MT conceived the study. All authors actively took part in the process. AD, JT, and MT planned and participated in the statistical analysis. All authors read and approved the final manuscript.

### Conflict of Interest Statement

The authors declare that the research was conducted in the absence of any commercial or financial relationships that could be construed as a potential conflict of interest.
